# Application of Scan Statistics to Detect Suicide Clusters in Australia

**DOI:** 10.1371/journal.pone.0054168

**Published:** 2013-01-14

**Authors:** Yee Tak Derek Cheung, Matthew J. Spittal, Michelle Kate Williamson, Sui Jay Tung, Jane Pirkis

**Affiliations:** 1 School of Public Health, The University of Hong Kong, Hong Kong, China; 2 Centre for Health Policy, Programs and Economics, Melbourne School of Population Health, The University of Melbourne, Melbourne, Australia; 3 Department of Earth Science, The University of Hong Kong, Hong Kong, China; Kenya Medical Research Institute - Wellcome Trust Research Programme, Kenya

## Abstract

**Background:**

Suicide clustering occurs when multiple suicide incidents take place in a small area or/and within a short period of time. In spite of the multi-national research attention and particular efforts in preparing guidelines for tackling suicide clusters, the broader picture of epidemiology of suicide clustering remains unclear. This study aimed to develop techniques in using scan statistics to detect clusters, with the detection of suicide clusters in Australia as example.

**Methods and Findings:**

Scan statistics was applied to detect clusters among suicides occurring between 2004 and 2008. Manipulation of parameter settings and change of area for scan statistics were performed to remedy shortcomings in existing methods. In total, 243 suicides out of 10,176 (2.4%) were identified as belonging to 15 suicide clusters. These clusters were mainly located in the Northern Territory, the northern part of Western Australia, and the northern part of Queensland. Among the 15 clusters, 4 (26.7%) were detected by both national and state cluster detections, 8 (53.3%) were only detected by the state cluster detection, and 3 (20%) were only detected by the national cluster detection.

**Conclusions:**

These findings illustrate that the majority of spatial-temporal clusters of suicide were located in the inland northern areas, with socio-economic deprivation and higher proportions of indigenous people. Discrepancies between national and state/territory cluster detection by scan statistics were due to the contrast of the underlying suicide rates across states/territories. Performing both small-area and large-area analyses, and applying multiple parameter settings may yield the maximum benefits for exploring clusters.

## Introduction

In the past decade, many countries have focused efforts on detecting and monitoring suicide clusters [1], [2], [3], [4], [5]. Suicide clusters are identified by the occurrence of a greater number of deaths by suicide than would be normally expected in a particular location and/or time period (i.e. the observed suicide rate is exceptionally higher than the expected rate in the underlying population). The presence of suicide clusters is generally explored in space only – that is, the geographic variation in deaths is of primary interest and time is held constant. In some cases, however, the incidence of suicide resembles a slowly developing epidemic, and it is more appropriate to consider the variation in time also. Suicide by charcoal-burning in several Asian countries is an example of this [6]. From an initial, widely publicized suicide in 1998, charcoal-burning became the second most common method in Hong Kong and Taiwan within 5 years. The detection of suicide clusters is important from a suicide prevention perspective because it allows gatekeepers, including medical professionals and social workers, to identify potential high-risk areas and to intervene to potentially avert suicide deaths and injuries from attempted suicide. The early identification of clusters may also aid postvention strategies that seek to minimize suicide contagion.

In Australia, the suicide rate increased modestly during the 1980s, reaching a peak in the late 1990s, where it began to decline steeply [7]. This trend was exacerbated for indigenous people [8]. The Northern Territory now has one of the highest suicide rates in the world, with a male suicide rate of 35.6 per 100,000 between 1998 and 2007 [9]. (In contrast, the overall Australian suicide rate in 2010 is 10.5 per 1000,000 [10].) Geographic studies have identified areas of high risk, and by extension, areas where suicide clusters may have occurred [5], [11]. Recent Australian studies have identified spatial clusters in the Northern Territory [12]. A study by Hanssens investigated suicide clusters in the Northern Territory and used a method known as the Knox test to verify the presence of suicide clustering [12]. A more recent study by Qi et al. (2012) examined evidence for spatial clusters, and identified a number of such clusters [5]. However, the broader picture of the epidemiology of suicide clustering remains unclear. For instance, there is as yet no agreed method for identifying clusters. A few studies have examined the rate of and risk factors for spatial clustering [3], [5], and the research about the epidemiology of spatial-temporal clustering based on a large scale of suicide data remains scarce.

The method that is commonly used for detecting disease clusters is the scan statistic [13]. The method applies moving windows scanning over the study area to explore possible clusters in space (spatial clustering) and time (temporal clustering). The method tests whether the number of cases within any spatial/temporal window exceeds the number expected by random process. The method has previously been applied to the identification of suicide clusters. Three previous suicide studies have detected clusters in the spatial dimension [2], [3], [5] and four studies have explored the temporal dimension [4], [14], [15], [16]. To the best of our knowledge no studies of suicide have simultaneously examined both dimensions with this methodology. Detection of spatial-temporal clusters has a different scope from spatial-only and temporal-only analysis. Spatial-only analyses have tended to focus on describing the spatial pattern of mortality and its relationship to area deprivation. Temporal analysis focuses on trends or peaks of mortality in one aggregated area over a period of time. Spatial-temporal cluster refers to an outbreak in a small region of the whole study within a short time frame, which are more related to the emergence of clustering and contagion. Detection of spatial-temporal clusters offers the potential to explore the factors underlying clustering, and help consolidate postvention strategies.

One of the limitations of using scan statistic is that the results are typically sensitive to the parameter settings in running the statistical program SaTScan [17]. For instance, modifications to size of the area under investigation, the number of iterations used to compute the solution, the maximum sizes of spatial and temporal windows, and unit of time and space have all been shown to alter the location of clusters [18], [19], [20]. To date, most previous research into the detection of suicide clusters has only sought to examine clusters at the national level. That is, clusters can potentially exist across state boundaries, but this approach may be insensitive to clusters that are occurring at a more fine-grained level. We broadened this research by examining the occurrence of suicide clusters within states/territories of Australia, by conducting the scan statistics in each state/territory separately. We use the population aged 10 and above as the denominator. The results were then compared with those from national cluster detection, which were obtained from conducting a similar analysis for the whole country.

## Methods

### Ethics Statement

The study was approved by the Human Research Ethics Committee of the Victorian Department of Justice, Australia.

### Study Design

The research design comprised a population-based retrospective study of all completed suicides which occurred in Australia. The postcode of residence and incident date of the suicide for each suicide case were used for space and time aggregation. Based on a Poisson Discrete model of scan statistics [13], the cluster detection attempted to identify which postal areas and time periods formed statistically significant suicide clusters.

### Suicide Data

Archival data on completed suicides occurring from 2004 to 2008 in Australia were obtained from the National Coroners Information System (NCIS), a database of all deaths in Australia certified by the coroner. The database records the date of death, cause of death, postal address of the deceased and a range of other variables relevant to the death investigation. We initially extracted 10,616 records from the database where the intent type was coded as intentional self harm (ICD 10: X60–X84) and the suicide incident occurred between 2004 and 2008. From this, 440 cases were excluded from the analysis due to missing date of death information (346 cases) or missing or incorrect postcode data (94 cases). Missing data for these 94 cases was due to the decreased being homeless, missing residential information in the coronial system, or invalid address that cannot be geocoded and mapped on the spatial map. As a result, 10,176 suicide cases (95.9%) that had complete and reliable information about the location and date of incidents were available for cluster detection analysis.

### Geographic Data

Population estimates of the number of individuals aged 10 and above in each postal area were obtained from the 2006 census data of Australian Bureau of Statistics. Digitized maps for each state/territory for the same period were also obtained from the Australian Bureau of Statistics. These maps were merged with the ArcGIS (version 9.0) to form a digital boundary map file containing 2,507 postal areas in Australia. The coordinates of the centroids of all postal areas were computed with the ArcGIS software. All suicide cases that occurred in a postal area were aggregated in the corresponding centroid.

### Statistical Analysis

The underlying principle of scan statistics is the use of a cylindrical window with a circular geographical base, and a height corresponding to time, that moves across the study space to detect clusters. If *G* is the whole space, and *n* is the total number of events (i.e. suicide cases) in the space *G*, as the cylindrical window moves over *G*, it defines a collection of windows *W*. Each *W* denotes a potential cluster that circles the centroids representing the census districts. The analysis compares the observed number of events, *n*(*W*) to the expected number of events, *e*(*W*) with a greater than expected number of events providing evidence of a cluster. The parameter of interest is λ, the likelihood function representing the space-time scan statistics, which is defined as.
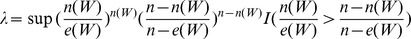



*I*() is an indicator variable with value 1 when the cylinder has more cases than expected under null hypothesis and 0 otherwise. Under the null hypothesis, the expected number of cases in each potential cluster is proportional to the population size of the cluster [21]. The expected number of events in each window is estimated with indirect standardization and covariate adjustment [22]. If *c_i_* is the observed number of events in the *i*th covariate category for each window and *p_i_* the corresponding population size then *C_i_* and *P_i_* are the total number of events and population of the *i*th covariate category in space G. The adjusted expected number is then calculated by:
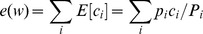



Single centroid points represent each postal area, where cases in each area are aggregated together [21]. If the circular base of the scanning window contains the centroid of a postal area, then the cases of the corresponding postal area are included in the window [23].

The scanning process identifies a zone from the data that is most likely to be a cluster, where the likelihood function, λ, can be maximized [13]. The likelihood ratio is complied by dividing the maximized likelihood function by the likelihood function with null hypothesis [13]. The statistical significance of the cluster is then evaluated with Monte Carlo testing by simulating 999 replications of the data set (giving 1,000 datasets when the observed dataset is included). For each simulated data set, the likelihood ratio of the most likely cluster is calculated in the same manner as that for the real data set. The probability that the expected number of events differ from the observed number of events in the most likely cluster is obtained through comparing the rank of the maximum likelihood function from the real data set with the likelihood ratios from the simulated data sets. Thus
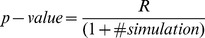
where *R* is the rank of maximum likelihood ratio from the real data set.

The software SaTScan is used for conducting the spatial-temporal cluster detection and testing the significance of clusters. In each scan, the maximum size of the spatial and temporal window is defined by the user. Scanning windows from the smallest size to this maximum are applied during the scanning process. Millions of windows with varying radius of the circular base, representing the geographical space, and varying height, representing the time, are generated in each run. These parameters not only set up the maximum sizes of the scanning window for both the true and simulated data, but also influence the critical levels for testing the possible clusters. The user must define the optimal values of these parameters, and in practice, the default values of 10%, 20% and 50% of the population-at-risk are often used. The reliance on these default values introduces subjectivity into the process and may hinder the identification of other clusters. Considering a wider range population-at-risk values is one way of overcoming this problem.

We used these methods to undertake two analyses to detect suicide clusters in Australia. One analysis was at the national level; the other at the state/territory level. For both analyses, the maximum temporal window parameter was fixed at 1, 2 and 3 months. For each value of maximum temporal window, the maximum spatial window parameter was set from 1% to 50% of the population at risk. We undertook 150 scans for the national analysis. The data was then split into the eight states and territories in Australia for cluster detection within each jurisdiction. A total of 150 scans were applied for each state/territory. We list all significant clusters (*p*<0. 05) found in national analysis and in the state/territory analysis. We examined similarities and differences between the two analyses, and compared their output statistics (expected frequencies and log-likelihood ratios) to explain the differences between the two cluster detections.

## Results

### Suicides in Australia

Suicide in Australia had a declining trend, from 2198 cases in 2004 to 1824 cases in 2008 (**Table 1**). More than three quarters of suicides were males (77.9%). The most common method of suicide was hanging (47.2%). The three states/territories with the highest proportion of suicides were New South Wales (25.2%), Queensland (23.9%), and Victoria (22.6%).

**Table 1:** Characteristics of all the suicides in Australia (2004–2008).

Characteristics		n	Percent
Gender	Female	2249	22.1
	Male	7927	77.9
Age group	Below 20	533	5.2
	20–39	4009	39.4
	40–59	3711	36.5
	60 or above	1921	18.9
	Unknown	2	0.0
Marital status	Married (inc de facto)	3593	35.3
	Divorced	616	6.1
	Never Married	2658	26.1
	Separated	987	9.7
	Widowed	343	3.4
	Unknown	1979	19.4
Employ	Employed	4213	41.4
	Unemployed	2001	19.7
	Retired/Pensioner	2227	21.9
	Student	409	4.0
	Home Duties	214	2.1
	Others	1112	10.9
Aboriginal origin	Neither Aboriginal nor TSI	7808	76.7
	Aboriginal Not TSI	417	4.1
	TSI Not Aboriginal	4	0.0
	Both Aboriginal and TSI	17	0.2
	Still Enquiring	1	0.0
	Unknown	1928	18.9
Year of incident	2004	2198	21.6
	2005	2125	20.9
	2006	2054	20.2
	2007	1975	19.4
	2008	1824	17.9
Suicide method	Hanging	4803	47.2
	Carbon monoxide poisoning	1281	12.6
	Drug poisoning	1228	12.1
	Firearm	807	7.9
	Transport injury	397	3.9
	Falling from height	488	4.8
	Drowning	177	1.7
	Fire or burn	126	1.2
	Cutting or stabbing	220	2.2
	Other ways of obstruct breathing	219	2.2
	Others	103	1.0
	Unknown	327	3.2
Residential state	Australian Capital Territory (ACT)	157	1.5
	New South Wales(NSW)	2565	25.2
	Northern Territory(NT)	205	2.0
	Queensland (QLD)	2431	23.9
	South Australia (SA)	943	9.3
	Tasmania (TAS)	370	3.6
	Victoria (VIC)	2303	22.6
	West Australia (WA)	1202	11.8

### National Cluster Detection

The national cluster detection with the maximum temporal scanning window of 1, 2 and 3 months and varying maximum spatial window identified 2, 8, and 7 significant suicide clusters respectively. (**Table 2**). Significant suicide clusters with higher numbers of observed suicide cases than expected contained 211 suicide cases, which comprised 2.1% of all suicides.

**Table 2:** Significant clusters identified by national cluster detection.

Maximum size of temporal window = 1 month								
Cluster ID	Locations	Time period	Observedcases	Expectedcases	Relativerisk	p-value	Log-likelihoodratio	Nested withinCluster
1A	SA	3/2004	3	0.00089	3370.06	0.002	21.36	Nil
1B	NT, QLD	11/2004	36	12.60	2.86	0.036	14.77	2E, 2F, 2G, 3B
**Maximum size of temporal window = 2 months**								
**Cluster ID**	**Locations**	**Time period**	**Observed** **cases**	**Expected** **cases**	**Relative** **risk**	**p-value**	**Log-likelihood** **ratio**	**Nested within** **Cluster**
2A	NT, QLD	1–2/2004	14	1.93	7.25	0.025	15.66	Nil
2B	SA	3/2004	3	0.00089	3370.06	0.002	21.37	Nil
2C	NT, QLD, WA	10–11/2004	71	28.06	2.54	0.001	23.06	Nil
2D	NT, QLD, WA	10–11/2004	67	26.16	2.57	0.001	22.25	Nil
2E	NT, QLD	10–11/2004	45	16.17	2.79	0.005	17.26	2C, 2D
2F	NT, QLD	10–11/2004	51	19.12	2.68	0.002	18.21	2C, 2D
2G	NT, QLD	10–11/2004	60	23.35	2.58	0.002	20.04	2C, 2D
2H	WA	12/2005–1/2006	14	1.28	10.96	0.002	20.78	Nil
**Maximum size of temporal window = 3 months**								
**Cluster ID**	**Locations**	**Time period**	**Observed** **cases**	**Expected** **cases**	**Relative** **risk**	**p-value**	**Log-likelihood** **ratio**	**Nested within** **Cluster**
3A	SA	3/2004	3	0.00089	3370.06	0.002	21.37	Nil
3B	QLD	11/2004–1/2005	73	35.37	2.07	0.042	15.34	Nil
3C	QLD	11/2004–1/2005	76	36.22	2.11	0.021	16.62	Nil
3D	SA	3–5/2005	24	5.72	4.20	0.013	16.15	Nil
3E	WA	12/2005–1/2006	14	1.28	10.96	0.002	20.78	Nil
3F	NT, QLD	9–11/2007	26	4.60	5.66	0.001	23.66	3F
3G	NT, QLD, SA, WA	9–11/2007	36	8.20	4.40	0.001	25.49	Nil

SA – South Australia; NT- Northern Territory; QLD – Queensland; WA – Western Australia.

**Figure 1:**
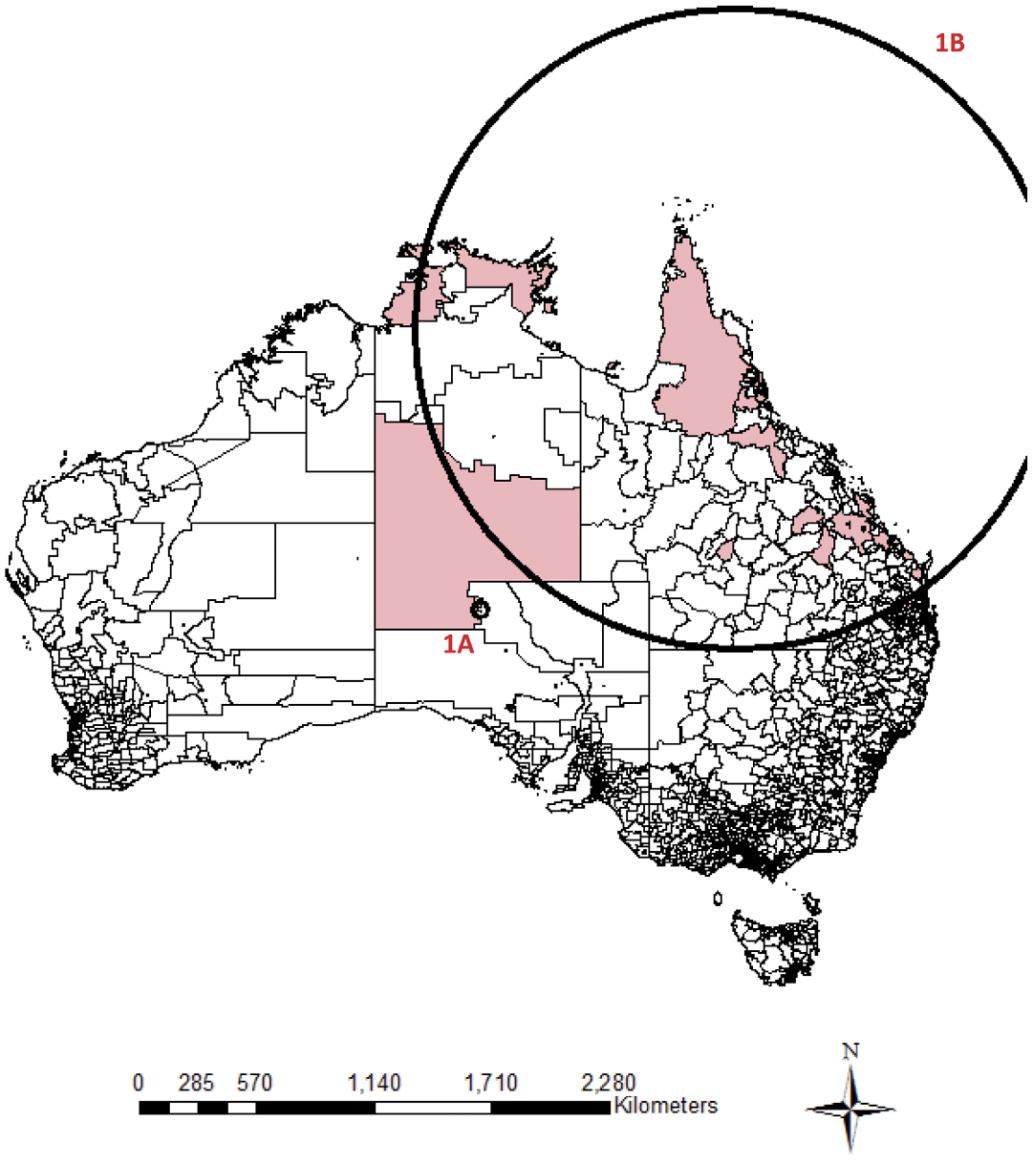
Significant clusters identified by national cluster detection with 1 month maximum temporal cluster size. Remark: Shaded regions are postal areas with completed suicide incidents within the particular clustering period. Circles represent the significant clustering windows detected by scan statistics.

The clusters were mainly located in the Northern Territory, northern Queensland, northern Western Australia and South Australia (**Figures 1** **,** **2** **and** **3** **,** **Table 2**). Almost no clusters were located close to coastal urban cities. The cluster size for the positive clusters ranged from 3 to 76 deaths. Postal areas within cluster circles did not necessarily have a suicide within the specified clustering period, especially those relatively larger cluster circles. Overlapping of geographical locations and occurrence durations was observed between these clusters. Small clusters nested within bigger clusters were important as they contributed to the overall clustering phenomenon.

**Figure 2:**
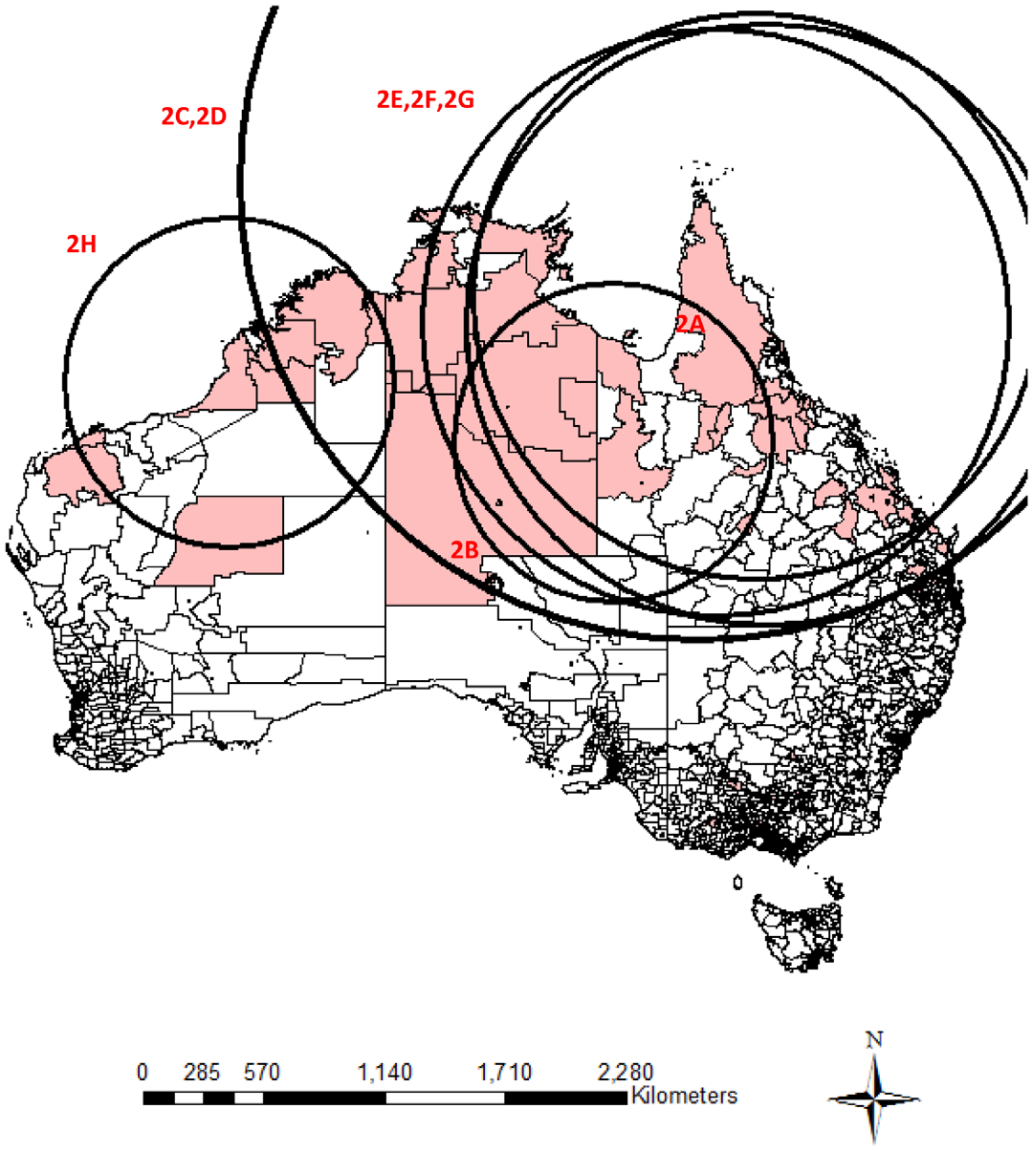
Significant clusters identified by national cluster detection with 2 month maximum temporal cluster size. Remark: Shaded regions are postal areas with completed suicide incidents within the particular clustering period. Circles represent the significant clustering windows detected by scan statistics.

**Figure 3:**
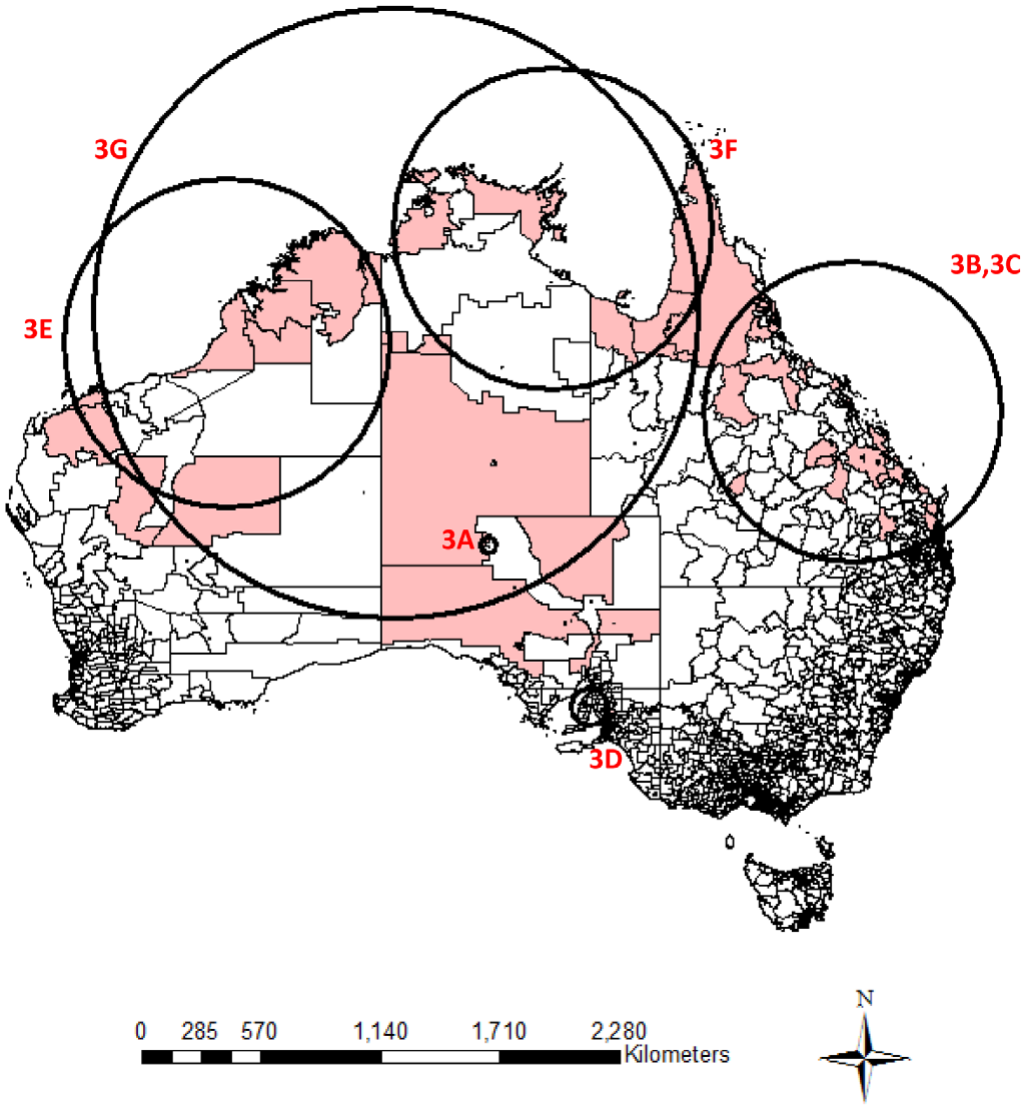
Significant clusters identified by national cluster detection with 3 month maximum temporal cluster size. Remark: Shaded regions are postal areas with completed suicide incidents within the particular clustering period. Circles represent the significant clustering windows detected by scan statistics.

### State/Territory Cluster Detection

**Figure 4:**
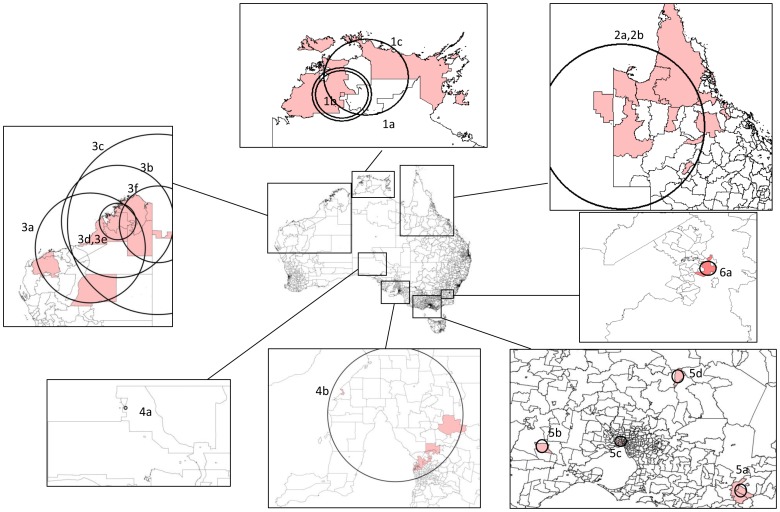
Significant clusters identified by state cluster detection.

The state cluster detection with the maximum temporal scanning window of 1, 2 and 3 months and varying maximum spatial window identified 5, 5 and 8 significant suicide clusters respectively. In total, 81 suicide cases in 18 clusters were distributed over the Northern Territory, Queensland, Western Australia, South Australia, Victoria and the Australian Capital Territory (**Table 3** **and** **Figure 4**). As with the national cluster detection, no significant clusters were found in New South Wales and Tasmania. The cluster sizes for the positive clusters were comparatively smaller, which ranged from 2 to 24 deaths. Combining both cluster detections, 243 suicides (2.4%) were identified as clustered suicides.

**Table 3:** Significant clusters identified by state cluster detection.

Locations	Cluster ID	Time period	Observedcases	Expectedcases	Relativerisk	p-value	Log-likelihoodratio	Nested withincluster
Northern Territory	1a	9/2007	2	0.022	90.31	0.044	7.02	1c
	1b	9–11/2007	8	1.13	7.36	0.046	8.93	1d
	1c	9–11/2007	11	1.88	6.13	0.007	10.53	Nil
Queensland	2a	1–2/2007	10	1.27	7.88	0.048	11.90	2b
	2b	1–3/2007	14	1.96	7.19	0.002	15.54	Nil
Western Australia	3a	12/2005	8	0.73	11.09	0.018	11.95	3b
	3b	12/2005–1/2006	10	0.80	12.55	0.001	16.06	3c
	3c	12/2005–1/2006	12	0.85	14.23	0.001	20.65	Nil
	3d	4/2006	4	0.083	48.31	0.014	11.59	3e
	3e	2–4/2006	6	0.25	24.46	0.008	13.41	Nil
	3f	12/2007–1/2008	6	0.28	21.29	0.012	12.62	Nil
South Australia	4a	3/2004	3	0.0011	2732.82	0.001	20.74	Nil
	4b	3–5/2005	24	6.87	3.56	0.028	13.04	Nil
Victoria	5a	7–9/2004	4	0.063	64.07	0.045	12.66	Nil
	5b	9–11/2004	3	0.012	242.79	0.021	13.49	Nil
	5c	11–12/2005	10	1.14	8.77	0.042	12.84	Nil
	5d	4/2006	2	0.0018	1103.98	0.037	12.01	Nil
Australian Capital Territory	6a	8–9/2004	2	0.015	137.23	0.011	7.85	Nil

### Comparison between National and State Cluster Detection

**Table 4** shows a summary of the national and state/territory cluster detection. As expected, the two detection methods had several consistent findings, such that both of them detected clusters in similar locations and time periods. Four clusters in Victoria found by the state/territory cluster detection were identified as non-significant clusters with the national cluster detection. Some clusters with smaller spatial sizes (2 in Western Australia, 1 in Queensland and 1 in Australian Capital Territory) were found by the state/territory cluster detection, but they were not identified by the national cluster detection. Meanwhile, clusters in relatively large spatial size or located across two or more states were only detected by the national cluster detection.

**Table 4:** Summary of suicide cluster streams from national and state cluster detection.

Time period	Locations	Clusters found by national cluster detection	Clusters found by state cluster detection
1–2/2004	NT & QLD	2A	–
3/2004	SA	1A, 2B& 3A	4a
9/2004	ACT	–	6a
7–9/2004	VIC	†	5a
9–11/2004	VIC	†	5b
10–11/2004	NT, QLD, SA & WA	1B, 2C, 2D, 2E, 2F, 2G	–
11/2004–1/2005	QLD	3B, 3C	–
3–5/2005	SA	3D	4b
11–12/2005	VIC	†	5c
12/2005–1/2006	WA	2H & 3E	3a, 3b,3c
4/2006	WA	–	3d
4/2006	VIC	–	5d
1–3/2007	QLD	–	2a
9–11/2007	NT, QLD, SA & WA	3F, 3G	1a, 1b, 1c
12/2007–1/2008	WA	–	3e

SA – South Australia; NT- Northern Territory; QLD – Queensland; WA – Western Australia; ACT – Australian Capital Territory.

† Found as insignificant clusters in national cluster detection.

**Figure 5:**
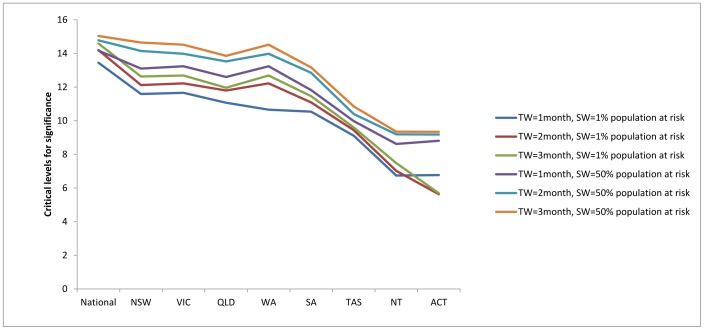
Critical levels for cluster significance (p<0.05) by national and state cluster detection. Remark: TW = Maximum temporal window size; SW = Maximum spatial window size.

The range of critical values for the national cluster detection used to determine the cluster significance is 13.5 to 15.0 (**Figure 5**). New South Wales, Victoria, Queensland and South Australia had similar ranges (11.1 to 14.7). South Australia and Tasmania have relatively lower ranges, which were 10.5 to 13.2 and 9.1 to 10.9, respectively. Northern Territory has the lowest range of critical values (6.7 to 9.4). These values suggest that the national cluster detection had higher critical values for determining cluster significance than all other state cluster detections. There were some differences in the critical values across the states/territories, and the Northern Territory and Australian Capital Territory had the lowest set of critical values. These differences precipitate the different findings from the two cluster detections.

Comparing the log-likelihood ratios of the detected clusters between the national and state cluster detection, only those clusters where same locations and time were detected by both cluster detection could be directly compared. The log-likelihood ratios between cluster 1A (expected cases = 0.00089, log-likelihood ratio = 21.36) and 4a (expected cases = 0.0011, log-likelihood ratio = 20.74) located in South Australia, and between 2H (expected cases = 1.28, log-likelihood ratio = 20.78) and 3c (expected cases = 0.85, log-likelihood ratio = 20.65) located in Western Australia did not vary greatly. On the other hand, the difference of log-likelihood between cluster 3D (expected cases = 5.72, log-likelihood ratio = 16.15) and 4b (expected cases = 6.87, log-likelihood ratio = 13.04) located in South Australia were comparatively larger. The aforementioned clusters in Victoria had smaller log-likelihood ratios in the national cluster detection than in the state cluster detection (values not shown), so they were not significant in the national cluster detection. These comparisons showed that the expected numbers of cases in the cluster detected by both cluster detections were not necessarily the same. Change of the study area results in the change of statistical significance.

## Discussion

### Key Results

Few epidemiological studies have applied spatial-temporal scan statistics to both national (combined states) and single-state level data to explore suicide clustering. Recent clustering studies have explored the presence of spatial clusters in Australia using only a fixed time period [2], [5], [11]. Two older studies [1], [12] tested for the presence of spatial-temporal clusters using the Knox method but neither study used any visual inspection techniques to identify the locations of the clusters [1], [12]. The current study explored the locations where suicide cases occurred in close spatial and temporal proximity. This type of clustering is more relevant to understanding the occurrence of point clustering and contagion [24]. No studies that examined suicide clustering with scan statistics adopted the flexible parameter settings that we used here. Previous studies often used defaulted values (e.g. 50% of the population-at-risk) as the maximum size of the scanning window. This study used a flexible parameter setting so that low likelihood clusters occurring within more likely clusters would be detected. In addition, the current study used a finer spatial unit (i.e. postcode, n = 2,507) for the analysis of clustering than Qi et al. [5] which used statistical local area as the spatial unit (n = 1,346).

Our study found evidence of a number of suicide clusters in Australia over the 2004–2008 period. Analysis at the national level identified the presence of two clusters over a one-month period involving 36 deaths (where 13 deaths would have been expected during the period if the number of deaths were in line with the size of the population aged 10 and older). The largest cluster was located in the Northern Territory and northern Queensland – all areas with large indigenous populations. Analysis using scanning windows with longer time periods identified additional clusters in other parts of the country but the presence of these large clusters in northern Australia persisted.

Analysis of the data at the state/territory level revealed significant clusters in all states except Tasmania and New South Wales. The largest discrepancies between the observed number of deaths and the expected number were in the Northern Territory and Western Australia. We identified 3 clusters comprising 11 deaths (but only 2 expected) in the Northern Territory and 6 clusters of 24 deaths (1.38 expected) in Western Australia. There was also a significant cluster in the remote northern part of the Queensland. Here there were 14 deaths where 2 would have been expected. We identified 4 additional clusters in Victoria that are noteworthy. In Victoria, 3 of the 4 clusters were located in regional areas. In all four were 19 deaths in areas where only 1 death would have been expected. In South Australia, one cluster was located close the capital city, Adelaide and comprised of 24 deaths (7 expected); the other was located in a regional area and comprised 3 deaths (less than 1 expected during the period). Lastly, a cluster in the Australian Capital Territory, where 2 suicides were involved, was detected (less than 1 expected during the period).

### Interpretation

In general, most of the suicide clusters were located in sparsely populated areas where the level of urbanization was low. Previous research has documented the high rates of suicide among indigenous Australians, particularly those living in the Northern Territory. For instance, in the 2001–2005 period, the age-standardized suicide rate in the Northern Territory was more than double the national rate and this finding has been attributed to the high number of indigenous deaths in the state [25]. This study extends these results by showing that suicide clusters are more likely to occur in areas where there is a high proportion of indigenous Australians. By implication, those who died in clusters were likely indigenous Australians. A past case study on indigenous suicide in the Northern Territory revealed that this group of people are more prone to suicide contagion than non-indigenous people due to denser social networks and interpersonal relationships with family and community [26]. Our findings provided some empirical support for this hypothesis.

These finding are also consistent with previous research that has examined the association between socio-economic deprivation and suicide clustering [3]. Our cluster detections found that most suicide clusters were located in some sparsely-populated inland areas of Queensland, Western Australia, and the Northern Territory. The locations of these clusters were remote areas which had a higher degree of socio-economic deprivation.

This study used two approaches to identify significant clusters – an analysis using national data and separate state/territory analyses. While there were a number of similarities between the results, a number of discrepancies arose also. These discrepancies can be explained by the change of expected case numbers, the log-likelihood ratios of the detected clusters and the critical values of scan statistics for determining the significance.

These results, however, do not, provide clear guidance as to which method of cluster detection is superior (i.e. national vs. state). Rather, the results suggest that the research question of interest should guide the focus of the cluster detection. Generally, the national cluster detection is capable of detecting larger, cross-state clusters, as it had a much larger size of the moving spatial window. Regional clusters, comprising of higher population-at-risk, are easier to be detected in national cluster detection with its greater statistical significance than community-level clusters.

The results of the current study are consistent with an earlier scan statistic study that modified the area under investigation [17]. Based on the evidence that the national and state/territory cluster detections yielded different expected suicides for some clusters, we found that changing the investigated area can influence the overall case rates and hence influence the outcome statistics. Gregorio et al. found that the discrepancy between combined-state and single-state cluster detection can be reduced by using a more restrictive parameter setting. In the current study, however, the consistency between the two levels is low, even though a more restrictive design with smaller sizes of spatial and temporal window has been used. A possible explanation is that the differential among the case rates of prostate cancer of the three states examined in Gregorio et al. was small. We observed from their study that the expected case number of the clusters in their study did not change much from combined-state to single-state analysis. On the contrary, the suicide rates across the states/territories in Australia were more heterogeneous. The difference of case rate across the nation and different states/territories in Australia was rooted from the differential of suicide risk between urban and rural areas, and across the eight states/territories, which have been supported in previous studies [9], [27]. Some of the expected case numbers and the log-likelihood ratios of the detected clusters of the national and state/territory cluster detection differed to a greater extent. Therefore, the statistical significance for clusters between the two cluster detections cannot be consistent. In other words, the discrepancy between national and state/territory cluster detection cannot be fully resolved by restricting parameter setting in the case of varying case rates across states/territories.

Another implication of our findings is that the strength of closeness of a cluster does not only depend on the closeness between incidents within cluster, but also the closeness and risk extent of incidents outside the potential cluster. As rural/remote areas and some states including Northern Territory, Tasmania, and Queensland have elevated suicide risk, the overall suicide rate is higher in the national cluster detection. For instance, Victoria had a lower suicide rate than the overall Australia figure. Some suicide incidents in Victoria were detected as having sufficient closeness to form a cluster from the analysis with the Victoria’s data, but cannot be detected as significant cluster in the national cluster detection. This phenomenon implies that the strength of closeness of a cluster does not only depend on the closeness between incidents within cluster, but also the risk extent outside the potential cluster. We suggest that, considering that cluster detection with scan statistics is sensitive to the underlying differential of spatial suicide risk, both national and state cluster detection would be needed to capture all possible clusters for users of SaTScan.

### Study Limitations

Our study had some methodological limitations which must be acknowledged. We used geographical and temporal proximity as the dimensions for determining clustering; however, there are other aspects of proximity that could determine clustering, for instance interpersonal or familial proximity. Thus, related suicides that are many months apart or suicides in a familial group that are across vast geographical distances might have been overlooked in our cluster detection. Detecting clusters related to familial or filial proximity was out of the current study scope. This is likely to be negligible in our study as we can assume close families or peers may live in the same or close postal areas.

Second, because the study applied only a cylindrical space-time scan statistic, non-circular clusters cannot be identified. A flexibly shaped space-time scan statistics would have some advantages in detecting irregularly clustering areas [28], [29]. Takahashi et al. (2008) has found that the cylindrical scan yielded comparable sensitivity and positive predictive value with the flexibly shaped scan, except for some extremely irregular cluster shapes [29].

Third, a drawback of using postal areas is that postal areas in Australia differ with respect to geographic and population size. Postal areas in inland areas are generally small and have a smaller population density (especially in remote postcodes). This results in very large spatial distances between some postal area centroids in these inland areas. Yet the spatial size of the Northern Territory, with 1% of the population at risk, exerts strong influence in national cluster detection. This explains why some clusters located in those inland areas are extraordinarily large. In addition, the postcodes in some clusters had small population size (e.g. 1A in Table 2) or short duration of clustering (e.g. 5b in Table 3), so had a very small number of expected cases. The relative risk for these clusters should be interpreted cautiously.

From the statistics of the monthly pattern of suicide for all the states/territories (table not shown), we found that weak evidence for a seasonal suicide pattern. Suicides were more frequent in January, October and November. We believe that seasonal pattern may have a considerable impact on the clustering findings, such that some big clusters are often found in those months (e.g. cluster 2C and 3G). However, we did not perform the adjustment on the following grounds: (1) Seasonal adjustment may over-adjust the temporal trend of suicides, and lead to overlooking of some original clusters; (2) Even without adjustment for seasonality, clustering in non-peak months can still be detected, such as cluster 1A and 3D. Nevertheless, the temporal trend could be adjusted for in future studies.

### Conclusion

This study has attempted to improve geo-statistical techniques for the detection of suicide clusters in Australia. The accurate identification of suicide clusters in a timely manner is important for postvention efforts to prevent possible contagion. Our findings illustrated that the majority of spatial-temporal suicide clusters were located in the inland areas with high levels of socio-economic deprivation and a high proportion of indigenous people.
